# Proteomic Profiling of the Human Fetal Multipotent Mesenchymal Stromal Cells Secretome

**DOI:** 10.3390/molecules25225283

**Published:** 2020-11-12

**Authors:** Arseniy A. Lobov, Natalia M. Yudintceva, Alexey G. Mittenberg, Sergey V. Shabelnikov, Natalia A. Mikhailova, Anna B. Malashicheva, Mikhail G. Khotin

**Affiliations:** Institute of Cytology of the Russian Academy of Science, 194064 St. Petersburg, Russia; arseniylobov@gmail.com (A.A.L.); yudintceva@mail.ru (N.M.Y.); a.mittenberg@gmail.com (A.G.M.); sergey.v.shabelnikov@gmail.com (S.V.S.); natmik@mail.ru (N.A.M.); h_mg@mail.ru (M.G.K.)

**Keywords:** MSCs, FetMSCs, mesenchymal stem cells, multipotent mesenchymal stromal cells, secretome, proteomics, regenerative biomedicine

## Abstract

Secretome of multipotent mesenchymal stromal cells (MSCs) is actively used in biomedical applications such as alveolar bone regeneration, treatment of cardiovascular disease, and neurodegenerative disorders. Nevertheless, hMSCs have low proliferative potential and production of the industrial quantity of their secretome might be challenging. Human fetal multipotent mesenchymal stromal cells (FetMSCs) isolated from early human embryo bone marrow are easy to expand and might be a potential source for pharmaceutical substances production based on their secretome. However, the secretome of FetMSCs was not previously analyzed. Here, we describe the secretome of FetMSCs using LC-MALDI shotgun proteomics. We identified 236 proteins. Functional annotation of the identified proteins revealed their involvement in angiogenesis, ossification, regulation of apoptosis, and immune response processes, which made it promising for biomedical applications. The proteins identified in the FetMSCs secretome are involved in the same biological processes as proteins from previously described adult hMSCs secretomes. Nevertheless, many of the common hMSCs secretome components (such as VEGF, FGF, Wnt and TGF-β) have not been identified in the FetMSCs secretome.

## 1. Introduction

Many human diseases come from the limits of tissue regenerative potential. Regenerative medicine focuses on the expansion of regenerative potential through control of tissue regeneration and cell proliferation/differentiation [1,2]. One of the promising approaches is the pluripotent embryonic stem cells (ESCs) treatment. ESCs might give rise to more than 200 tissues, some of which have no self-renewal capacities in an adult organism [3]. Nevertheless, ESCs have compromised genomic stability, which creates an obstacle to their use in therapy and increases the risk of carcinogenesis and immunologic rejection [4,5].

Multipotent mesenchymal stromal cells, on the other hand, have higher genomic stability, low carcinogenesis risk, and restricted proliferative potential, but might be differentiated into many cell types: bone, cartilage, adipose, skin, trachea, cornea, muscle, nerve, liver and myocardium [5,6]. Thus, MSCs are assumed to be a promising therapeutic option and have become the most frequently used stem cell type in clinical trials (a total of 9507 clinical trials were registered according to the ClinicalTrials.gov at the October of 2020).

MSCs are known to release a complex of paracrine factors that might modulate immune reactions and tissue regeneration [7,8]. Moreover, in many cases, their secretome demonstrates the same or higher therapeutic potential than MSCs itself–most of the clinical benefits of MSCs might be achieved by a cell-free therapy approach [2,7,8].

One of the main challenges in cell-free therapy is to obtain an easily expandable cell system, which might produce industrial amounts of pharmacological substances with predictable composition and biological properties. Despite the fact that the hMSCs might be easily isolated from various sources, including fat, blood and urine, obtaining a highly-expandable culture of these cells with stable molecular properties is challenging. MSCs and their secretome might vary in terms of donors, cell isolation sources, and isolation and cultivation procedures, all of which are difficult to standardize completely [9,10,11,12,13,14,15].

In this context, fetal multipotent mesenchymal stromal cells (FetMSCs) isolated from early human embryo seem to be a promising cell type having higher proliferative potential than adult tissue-derived hMSCs [16]. Stable culture of FetMSCs from early human embryo bone marrow was established and described at the Institute of Cytology of the RAS [17,18,19]. FetMSCs form a homogenous population of fibroblast-like cells with normal human karyotype 46, XY, and are similar to adult bone marrow-derived mesenchymal stromal cells immunophenotypes CD44^+^, CD73^+^, CD90^+^, CD105^+^, HLA-ABC^+^, CD34^−^ and HLA-DR^−^. As hMSCs from adult tissues, these cells have no human embryonic stem cell markers: TRA-1-60 and Oct-4 [17,18].

As with other MSCs types, FetMSCs secretome demonstrates biological activity and osteoinductive properties and could be used in bone regeneration [19]. Moreover, FetMSCs are easy to expand in the automatic cultivation system CompacT SelecT (Sartorius, Royston, UK) up to 12-14 passages [19]. Standardization is important for the cell feeding process. Nevertheless, while secretomes of hMSCs from adult tissues have been described in details at proteomics, transcriptomics and metabolomics levels, the secretome of FetMSCs has not been studied yet. Here, we describe the secretome of FetMSCs in the context of their possible therapeutic use.

## 2. Results

We identified 236 proteins, which were presented at least in two of four biological replicates analyzed by LC-MALDI shotgun proteomics (a list of identified proteins is represented in the Supplement Material Table S1).

**Figure 1:**
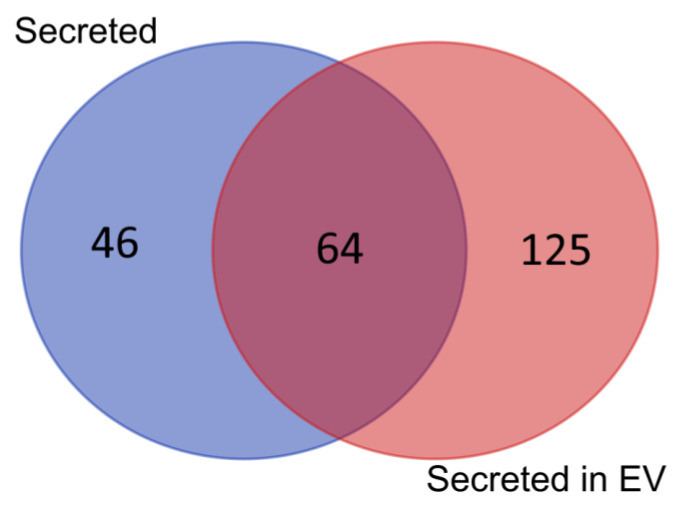
Venn diagram of the number of proteins identified in the fetal multipotent mesenchymal stromal cells (FetMSCs) secretome previously described as secreted (blue) or secreted in extracellular vesicles (EV; red) or secreted in both states (purple) based on UniProtKB annotation and information from Vesiclepedia database (www.microvesicles.org/) [20].

All identified proteins were previously described to be secreted as soluble proteins or to be secreted in extracellular vesicles, with exception of caveolae-associated protein 1 (Q6NZI2), which was not previously described as a part of cell secretome (Figure 1).

**Figure 2:**
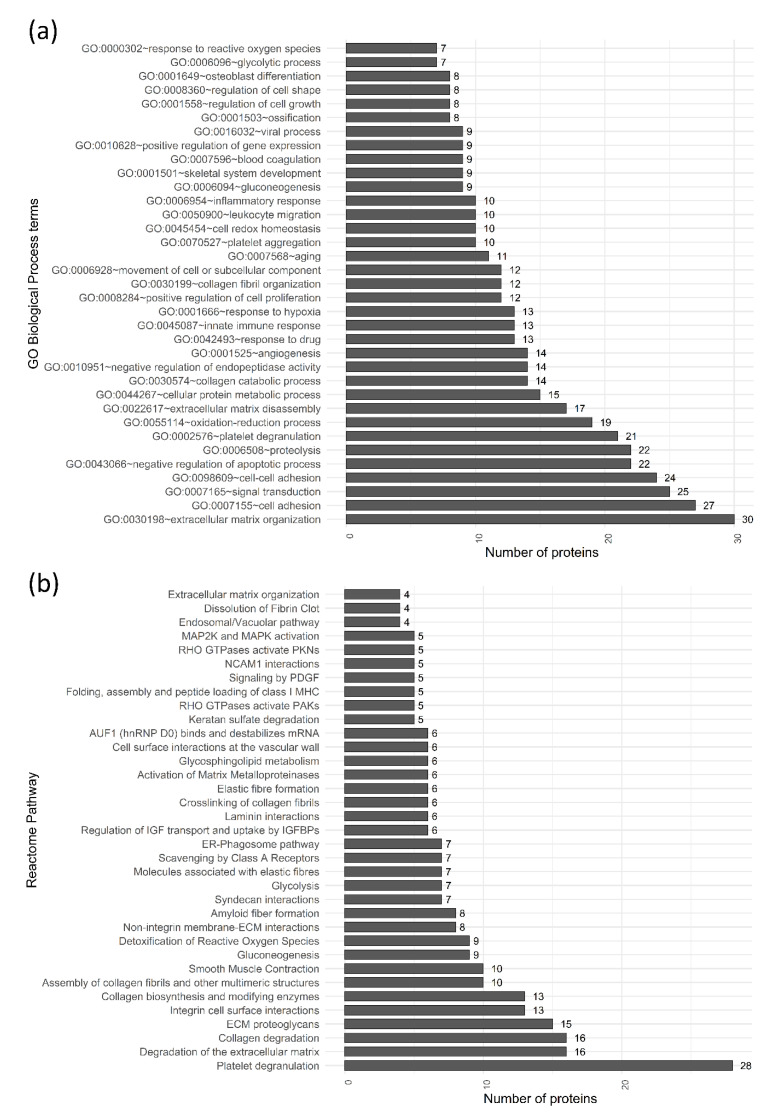
Bar charts representing functional annotation of proteins identified in the FetMSCs secretome by (**a**) gene ontology biological process and (**b**) reactome databases [21]. Top-35 categories are represented.

Enrichment analysis based on “gene ontology molecular function from publication” database revealed that 82% of the identified proteins are involved in binding of other molecules: protein binding (174 proteins; GO:0005515), poly(A) RNA binding (43 proteins; GO:0003723), calcium ion binding (37 proteins; GO:0005509), identical protein binding (31 proteins; GO:0042802), cadherin binding involved in cell-cell adhesion (29 proteins; GO:0098641), protein homodimerization activity (22 proteins; GO:0042803), integrin binding (18 proteins; GO:0005178), receptor binding (16 proteins; GO:0005102), etc. Detailed enrichment analysis by gene ontology (GO) biological process database and reactome database revealed that a significant part of the FetMSCs secretome is involved in therapeutically perspective biological processes (Figure 2).

**Figure 3:**
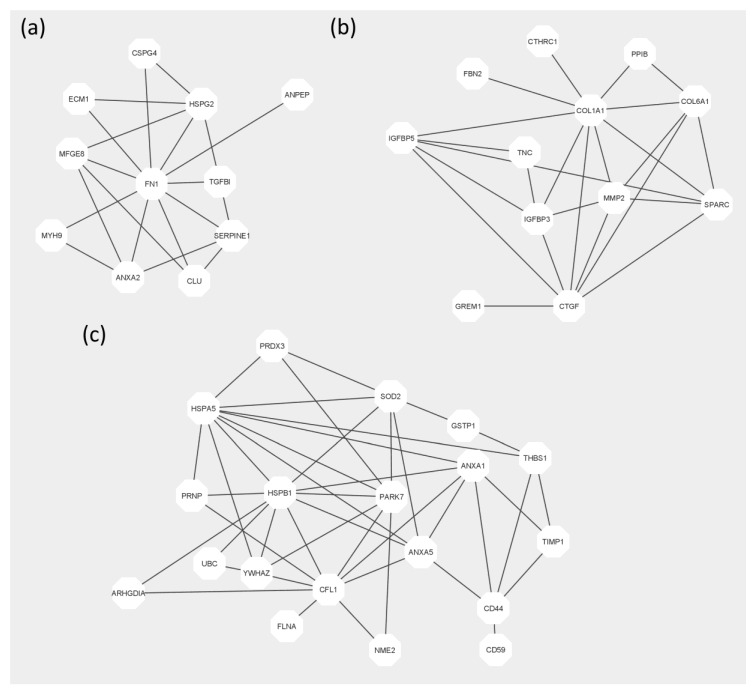
Interaction networks of proteins identified in the FetMSCs secretome involved in (**a**) angiogenesis, (**b**) osteogenesis and (**c**) negative regulation of apoptosis based on STRING database.

Proteins associated with angiogenesis (An), osteogenesis (Ost), ageing (Ag), apoptosis (Ap), platelet regulation (Plt) and regulation of immune response (IR) are listed in the SM Table S1. The interaction networks of FetMSCs secretome components probably involved in angio- and osteogenesis and apoptosis inhibition are shown in Figure 3.

Further, we compared the secretome of FetMSCs with those previously described for adult hMSCs and hESCs. For this purpose, we used published data of LC-MS/MS shotgun proteomics comparison of (1) three types of hMSCs isolated from adipose tissue (hADSCs), bone marrow (hBMSCs), and umbilical cord Wharton’s jelly (hWJSCs) [22], and (2) two hESCs lines: H9 and CA1 [23].

**Figure 4:**
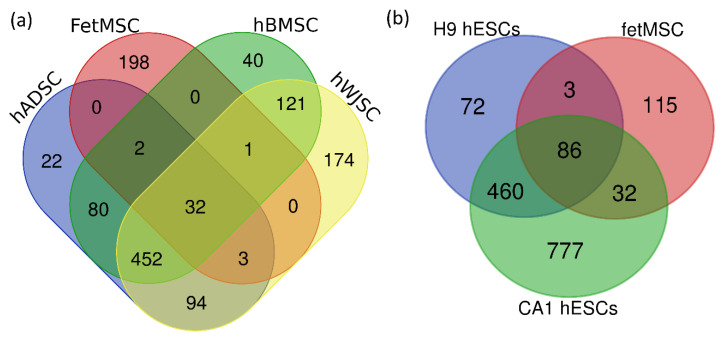
Venn diagram of the number of proteins shared between the FetMSCs secretome and previously published (**a**) three types of adult hMSCs secetomes [22] or (**b**) two types of hESCs secretomes [23].

The secretome of FetMSCs differs from both adult hMSCs and ESCs. Moreover, FetMSCs secretome shares more identical proteins with hESCs than with hMSCs (51% and 16% of similar proteins, respectively; Figure 4).

## 3. Discussion

Most biological processes were enriched in the FetMSCs secretome (Figure 2); “extracellular matrix organization”, “angiogenesis”, “immune response”, “ossification”, “osteoblast differentiation”, “negative regulation of apoptotic process”, and “leukocyte migration” were also enriched in secretomes of other MSCs-types [22,24]. At the same time, the similarity of FetMSCs secretome and some previously published secretomes of adult hMSCs is low (Figure 4). It is important to emphasize that some of these differences might be the result of dissimilarity in the cell culturing conditioning, but not in the cell type. Nevertheless, we have not identified many of the proteins usually presented in hMSCs secretome.

Particularly, key angiogenesis factors represented in hMSCs secretomes such as AKT1, FGF2, VEGF, Wnt3a, ANGPT1, von Willebrand factor, and TGF-*β* were not detected in the FetMSCs secretome. Nevertheless, the FetMSCs secretome contained other proteins known to positively regulate angiogenesis, such as extracellular matrix protein 1 [25], annexin A2 [26], aminopeptidase N [27], lactadherin [28] and connective tissue growth factor [29]. At the same time, there are two proteins known to inhibit angiogenesis: TGFBI [30] and plasminogen activator inhibitor-1 [31].

Classic osteogenic factors observed in the hMSCs secretomes were also absent in FetMSCs secretome. Nevertheless, we observed some other proteins involved in osteogenic differentiation: COL6A1 [32], MMP2 [33], tenascin [34], semaphorin-7A [35], stanniocalcin-1 [36], exostosin-1 and Exostosin-2 [37]. On the other hand, inhibitors of osteogenic differentiation were also observed: gremlin-1 [38], insulin-like growth factor binding protein-3 [39] and twisted gastrulation protein homolog 1 [40].

One of the major functional clusters enriched in the secretome was the one with proteins involved in negative regulation of apoptosis and immune response. The anti-apoptotic cluster included such known apoptosis inhibitors as filamin-A [41], CD 44 [42] and metalloproteinase inhibitor 1 [43]. Despite described anti-apoptotic effect of hMSCs secretome, this functional cluster is not usually enriched in the hMSCs secretome [44]. Nevertheless, the effect of immunomodulation repertoire of FetMSCs secretome cannot be predicted from protein identification data.

Proteins involved in the viral process represent another functional group enriched in FetMSCs secretome, which is not common for the adult hMSCs: fibulin-1, major histocompatibility complex, class I, C (HLA-C); heterogeneous nuclear ribonucleoprotein A1 (hnRNPA1), heat shock 70 kDa protein 8 (HSPA8), milk fat globule-EGF factor 8 protein (lactadherin), matrix metalloproteinase-1 (MMP-1), talin-1 and vimentin. Overexpression of fibulin-1 is known to inhibitpapillomavirus E6 protein-mediated transformation of cells [45]. HLA-C is an essential regulator of innate and antiviral immunity through its ability to interact with specific receptors in the NK-cells [46]. Moreover, the higher HLA-C surface expression is associated with slower HIV disease progression [47]. Both hnRNPA1 and HSPA8 have an ambiguous influence on viral infection: they can enhance replication of some viruses, but they decrease it for some others [48,49]. Lactadherin has been reported to bind specifically to rotavirus and inhibits its replication [50]. Talin-1 can act as a viral restriction factor that suppresses hepatitis B virus replication [51]. Vimentin is known to be involved in virus attachment and entry [52]. Thus, we assume that soluble vimentin might inhibit virus attachment. However, direct antiviral activity has not been described for any of these proteins, and possible antiviral effect of FetMSCs should be tested in the case of specific viruses.

Overall, we assume that angio- and osteoinductive properties of FetMSCs secretome might be lower than those of adult hMSCs secretome and might activate through different molecular mechanisms. Nevertheless, FetMSCs secretome may have higher anti-apoptotic effect and several other biological activities, which are not usual for secretomes of adult hMSC.

Finally, it is important to emphasize that we identified 239 of proteins presented at least in two replicates, but only 99 of them were presented in all four analyzed samples (SM Table S2, SM Figure S1). This is the result of high technical variation of used nanoLC-MALDI methodology. Moreover, we described the secretome of FetMSCs cell line isolated from one donor. Thus, the addition of technical and/or biological replicates from different donors might enhance the number of identified proteins and deepen the understanding of FetMSCs secretome variation.

## 4. Materials and Methods

### 4.1. Cell Culture

FetMSCs line was obtained from the Russian Collection of cell cultures of vertebrates (Institute of Cytology of the Russian Academy of Sciences, St. Petersburg, Russia) where it was deposited after its first characterization [17]. FetMSCs line was originally isolated from bone marrow of early human embryo and described as multipotent mesenchymal stromal cells based on the Minimal criteria of The International Society for Cellular Therapy [17,53]. The cells were cultured in Dulbecco’s Modified Eagle’s Medium (DMEM, Gibco by Life Technologies, Paisley, UK) containing 10% Fetal Bovine Serum (FBS, Gibco by Life Technologies, UK) and 1% Penicillin/Streptomycin (P/S, Gibco by Life Technologies, UK) at 37 °C in 5% CO_2_ until confluence of 80–90%. The cells from passages 3–5 were used in the experiments. FetMSCs were cultivated in four replicates.

### 4.2. FetMSCs Secretome Harvest

To remove the serum, the cells were washed six times with PBS preheated to 37 °C by shaking for 5 min. The number of washes was empirically optimized until the absence of serum albumin was obtained in the protein electrophoresis.

After washing, the FetMSCs were placed in a serum-free medium (DMEM, 1% P/S) and cultured under standard conditions for 48 h. After incubation, cell physiological state was estimated. Harvesting of MSCs secretome by cultivation in serum-free medium is a routine technique used elsewhere (e.g., [22]).

Conditioned medium (CM) was collected and immediately centrifuged at 500× *g* and at + 4 °C for 20 min to remove floated cells, then at 1500× *g* and at + 4 °C for 20 min to remove cell debris, and finally at 20,000× *g* and at + 4 °C for 20 min to remove small debris and huge extracellular vesicles. After each centrifugation, CM was transferred to a new tube. Resulting CM consisted of soluble extracted proteins and small extracellular exosomes.

### 4.3. Protein Isolation

The proteins were precipitated with four volumes of cold acetone, incubated overnight at −20 °C, and centrifuged at 20,000× *g* and at −10 °C for 20 min.

The protein pellets were dissolved in 50 mM ammonium bicarbonate buffer. For disulfide bound alkylation, the samples were consistently incubated in DTT (dithiothreitol; 10 mM, 80 °C, 15 min) and iodoacetamide (20 mM, RT, 30 min). Then, the protein precipitation with acetone was repeated and the pellets were dissolved in 50 mM ammonium bicarbonate buffer. The protein concentration was measured by QuDye Protein Quantification Kit (Lumiprobe, Hannover, Germany) according to the manufacturer instructions. Protein quality was checked by SDS-PAGE.

### 4.4. LC-MALDI MS/MS Analysis

A total of 15 ug of protein in 50 mM ammonium bicarbonate buffer was digested with 300 ng of Trypsin (1:50 ratio; Trypsin Gold, Promega, Madison, WI, USA) overnight at 37 °C. The digestion was stopped by the addition of TFA (trifluoroacetic acid) to 0.1% final concentration. The samples were dried in Martin Christ RVC-2-33IR rotary vacuum concentrator (Martin Christ, Osterode am Harz, Germany). Tryptic peptides were analyzed using nanoflow liquid chromatography coupled with MALDI-MS/MS.

Reverse-phase HPLC was performed using a Chromolith CapRod RP-18e HR column (0.1 mm × 150 mm, Merck, Darmstadt, Germany) in the Eksigent NanoLC Ultra 2D+ system (SCIEX, Darmstadt, Germany). Solvent A was 5% acetonitrile with 0.2% (*v*/*v*) TFA, and Solvent B was 60% (*v*/*v*) acetonitrile in water. The column was operated at a room temperature of 22–24 °C. The effluent from the column was mixed with matrix solution (α-Cyano-4-hydroxycinnamic acid 5 mg/mL, 0.2% (*v*/*v*) TFA in 95% methanol) containing two calibration standards bradykinin 2–9 (30 pM/mL) and ACTH 18-39 (60 pM/mL), at a flow rate of 1.4 μL/min. A micro-fraction collector was used to deposit 1 mm spots every 5 s, and a total of 1408 fractions was collected for each nano LC run. The column was washed with a gradient (0–100–100% B for 5 min and 2 min, respectively, at a flow rate of 800 nL/min) and equilibrated to 0% B for 3.5 min before subsequent injections.

The fractionated samples were analyzed with a TOF/TOF 5800 System (SCIEX, Darmstadt, Germany) instrument operated in the positive ion mode. The MALDI stage was set to continuous motion mode. MS data was acquired at 2600 laser intensity with 1000 laser shots/spectrum (200 laser shots/sub-spectrum), and MS/MS data were acquired at 3400 laser intensity with a DynamicExit algorithm and a high spectral quality threshold or a maximum of 1000 laser shots/spectrum (250 laser shots/sub-spectrum). Up to 25 top precursors with S/N > 40 in the mass range 750–4000 Da were selected from each spot for MS/MS analysis.

### 4.5. Protein Identification

The Protein Pilot 5.0 software (SCIEX, Darmstadt, Germany) with the Paragon algorithm 5.0 in thorough mode was used for the MS/MS spectra search against the UniProt human database. Carbamidomethyl cysteine was set as a fixed modification. False discovery rate (FDR) analysis was done by analysis of reversed sequences using the embedded PSEP tool.

Bioinformatics analysis was performed by functional annotation in DAVID functional annotation tool (6.8) [21] and Cytoscape (3.8) [54] with data visualization in R (3.6.1) [55,56].

## 5. Conclusions

Easy expandable human fetal MSCs cells are perspective for production of pharmacology active substances based on hMSCs secretome. FetMSCs secretome consists of 236 proteins, mostly unique to this cell type. Predicted biological activity of FetMSCs secretome seems to be similar to adult hMSCs. Nevertheless, the biological effect of FetMSCs secretome should be tested in the specific experimental conditions.

## Supplementary Materials

The following are available online at Table S1: Proteins, identified in FetMSCs secretome at least in two biological replicates, Table S2: Results of protein identification in four FetMSCs replicates. Figure S1: Venn diagram of identified proteins overlapping between biological replicates.
